# A spontaneous increase in intracellular Ca^2+^ in metaphase II human oocytes *in vitro* can be prevented by drugs targeting ATP-sensitive K^+^ channels

**DOI:** 10.1093/humrep/dev300

**Published:** 2015-12-18

**Authors:** Gonçalo Fernandes, Navin Dasai, Natalia Kozlova, Albaraa Mojadadi, Mandy Gall, Ellen Drew, Evelyn Barratt, Oladipo A. Madamidola, Sean G. Brown, Alison M. Milne, Sarah J. Martins da Silva, Katherine M. Whalley, Christopher L.R. Barratt, Aleksandar Jovanović

**Affiliations:** 1 Medical Research Institute, Ninewells Hospital and Medical School, University of Dundee, Dundee DD1 9SY, UK; 2 Department of Anatomy and Clinical Embryology, King Abdulaziz University, Jeddah, Saudi Arabia; 3 Assisted Conception Unit, NHS Tayside, Ninewells Hospital, Dundee; 4 University of Abertay, Dundee, UK

**Keywords:** human oocytes, Ca^2+^, ATP-sensitive K^+^ channels, pinacidil, glibenclamide

## Abstract

**STUDY QUESTION:**

Could drugs targeting ATP-sensitive K^+^ (K_ATP_) channels prevent any spontaneous increase in intracellular Ca^2+^ that may occur in human metaphase II (MII) oocytes under *in vitro* conditions?

**SUMMARY ANSWER:**

Pinacidil, a K_ATP_ channel opener, and glibenclamide, a K_ATP_ channel blocker, prevent a spontaneous increase in intracellular Ca^2+^ in human MII oocytes.

**WHAT IS KNOWN ALREADY:**

The quality of the oocyte and maintenance of this quality during *in vitro* processing in the assisted reproductive technology (ART) laboratory is of critical importance to successful embryo development and a healthy live birth. Maintenance of Ca^2+^ homeostasis is crucial for cell wellbeing and increased intracellular Ca^2+^ levels is a well-established indicator of cell stress.

**STUDY DESIGN, SIZE, DURATION:**

Supernumerary human oocytes (*n* = 102) collected during IVF/ICSI treatment that failed to fertilize were used from October 2013 to July 2015. All experiments were performed on mature (MII) oocytes. Dynamics of intracellular Ca^2+^ levels were monitored in oocytes in the following experimental groups: (i) Control, (ii) Dimethyl sulfoxide (DMSO; used to dissolve pinacidil, glibenclamide and 2,4-Dinitrophenol (DNP)), (iii) Pinacidil, (iv) Glibenclamide, (v) DNP: an inhibitor of oxidative phosphorylation, (vi) Pinacidil and DNP and (vii) Glibenclamide and DNP.

**PARTICIPANTS/MATERIALS/SETTINGS/METHODS:**

Oocytes were collected under sedation as part of routine treatment at an assisted conception unit from healthy women (mean ± SD) age 34.1 ± 0.6 years, *n* = 41. Those surplus to clinical use were donated for research. Oocytes were loaded with Fluo-3 Ca^2+^-sensitive dye, and monitored by laser confocal microscopy for 2 h at 10 min intervals. Time between oocyte collection and start of Ca^2+^ monitoring was 80.4 ± 2.1 h.

**MAIN RESULTS AND THE ROLE OF CHANCE:**

Intracellular levels of Ca^2+^ increased under *in vitro* conditions with no deliberate challenge, as shown by Fluo-3 fluorescence increasing from 61.0 ± 11.8 AU (AU = arbitrary units; *n* = 23) to 91.8 ± 14.0 AU (*n* = 19; *P* < 0.001) after 2 h of monitoring. Pinacidil (100 µM) inhibited this increase in Ca^2+^ (85.3 ± 12.3 AU at the beginning of the experiment, 81.7 ± 11.0 AU at the end of the experiment; *n* = 13; *P* = 0.616). Glibenclamide (100 µM) also inhibited the increase in Ca^2+^ (74.7 ± 10.6 AU at the beginning and 71.8 ± 10.9 AU at the end of the experiment; *n* = 13; *P* = 0.851. DNP (100 mM) induced an increase in intracellular Ca^2+^ that was inhibited by glibenclamide (100 µM; *n* = 9) but not by pinacidil (100 µM; *n* = 5).

**LIMITATIONS, REASONS FOR CAUTION:**

Owing to clinical and ethical considerations, it was not possible to monitor Ca^2+^ in MII oocytes immediately after retrieval. MII oocytes were available for our experimentation only after unsuccessful IVF or ICSI, which was, on average, 80.4 ± 2.1 h (*n* = 102 oocytes) after the moment of retrieval. As the MII oocytes used here were those that were not successfully fertilized, it is possible that they may have been abnormal with impaired Ca^2+^ homeostasis and, furthermore, the altered Ca^2+^ homeostasis might have been associated solely with the protracted incubation.

**WIDER IMPLICATIONS OF THE FINDINGS:**

These results show that maintenance of oocytes under *in vitro* conditions is associated with intracellular increase in Ca^2+^, which can be counteracted by drugs targeting K_ATP_ channels. As Ca^2+^ homeostasis is crucial for contributing to a successful outcome of ART, these results suggest that K_ATP_ channel openers and blockers should be tested as drugs for improving success rates of ART.

**STUDY FUNDING/COMPETING INTEREST(S):**

University of Dundee, MRC (MR/K013343/1, MR/012492/1), NHS Tayside. Funding NHS fellowship (Dr Sarah Martins da Silva), NHS Scotland. The authors declare no conflicts of interest.

## Introduction

Approximately 1 in 7 couples of reproductive age are classed as infertile, equating to ∼72 million people globally. The primary treatment is assisted reproductive technology (ART) consisting of IVF and ICSI. However, despite the rapid developments in ART over the last decade, current success rates remain both sub-optimal and variable. For example, in the European Union the average IVF pregnancy rate is 29.2% per aspiration—ranging from 21.5 to 48.1% (Kupka *et al.*, 2014). Even in oocyte donors, it is calculated that less than 7% of the oocytes produce a live birth—a figure that has shown little improvement in the last 12 years (Patrizio and Sakkas, 2009).

It is well established that the quality of the egg and maintenance of this quality during *in vitro* processing in the ART laboratory is of critical importance to successful embryo development and a healthy live birth (reviewed by Marteil *et al.*, 2009; Lord and Aitken, 2013). Additionally, there are recent reports suggesting that animals generated by IVF exhibit vascular dysfunction later in life and shortened lifespan in general. It has been proposed that stress associated with ART *in vitro* procedures is most likely responsible for this phenomenon (Rexhaj *et al.*, 2013). Removal of an egg from its natural environment (the follicle) and exposure to *in vitro* conditions is a considerable stress (Khosla *et al.*, 2001). Although there is wide variation in ART laboratory practice, following follicle aspiration eggs can be left for several (∼2–6) hours and, in the case of ICSI, stripped of their cumulus before injection/addition of sperm. Although handling and manipulation of the eggs in the laboratory aims to minimize external stresses, data from work on oocytes of experimental animals and many other cell types indicate that cellular stress might occur (Takahashi *et al.*, 2009). An important effect of *in vitro* stress is likely to be disturbance of ionic regulation of the cell. For instance, oxidative stress (H_2_O_2_) and *in vitro* ageing of metaphase II (MII) eggs in mice induces impairment of calcium homeostasis and poor subsequent embryo development (Takahashi *et al.*, 2009). Higher intracellular Ca^2+^ is observed with oxidative stress, and evidence of impaired store-operated calcium entry is found in *in vitro* aging (24 h) (Martín-Romero *et al.*, 2008). An increase in intracellular Ca^2+^ has been recognized as a trigger of events that ultimately lead to cell death. However, in all studies addressing Ca^2+^ homeostasis in oocytes *in vitro*, stress was induced by different means. Whether Ca^2+^ levels in oocytes remain steady under routine *in vitro* conditions when there is no deliberate induction of stress is yet unknown. Any change in Ca^2+^ dynamics has the potential to affect intracellular signalling and could be associated with unsuccessful fertilization and/or health issues later in life.

The regulation of Ca^2+^ homeostasis is crucial for cellular protection during stress (Clapham, 2007), and impaired Ca^2+^ homeostasis in oocytes negatively impacts fertilization and development (Miao *et al.*, 2009). ATP-sensitive K^+^ (K_ATP_) channels are K^+^-selective channels gated by intracellular ATP. As such, they are suggested to be a link between intracellular metabolic conditions and cellular membrane excitability. In tissues where they are expressed, K_ATP_ channels play a crucial physiological role, including a role in insulin secretion, appetite control, smooth muscle tone and others (reviewed by Nichols, 2006). Additionally, regulation of channel activity and numbers has been shown to regulate cellular resistance to different types of stresses in different types of cells (Crawford *et al.*, 2002; Mohammed Abdul *et al.*, 2014, 2015a,b). In oocytes, K_ATP_ channels have been recently identified (Du *et al.*, 2010), but their physiological role is unknown. It is also unknown whether drugs targeting K_ATP_ channels would have any effect on oocyte function. If Ca^2+^ homeostasis is challenged under routine ART laboratory conditions, oocyte intracellular signalling pathways may be sufficiently compromised so as to reduce the chances of successful conception without any external signs to indicate this. Therefore, the aim of the present study was to establish whether intracellular Ca^2+^ levels change in human oocytes during incubation *in vitro* and, if it does, would drugs targeting K_ATP_ channels counteract such changes.

## Materials and Methods

### Human oocytes

**Figure 1:**
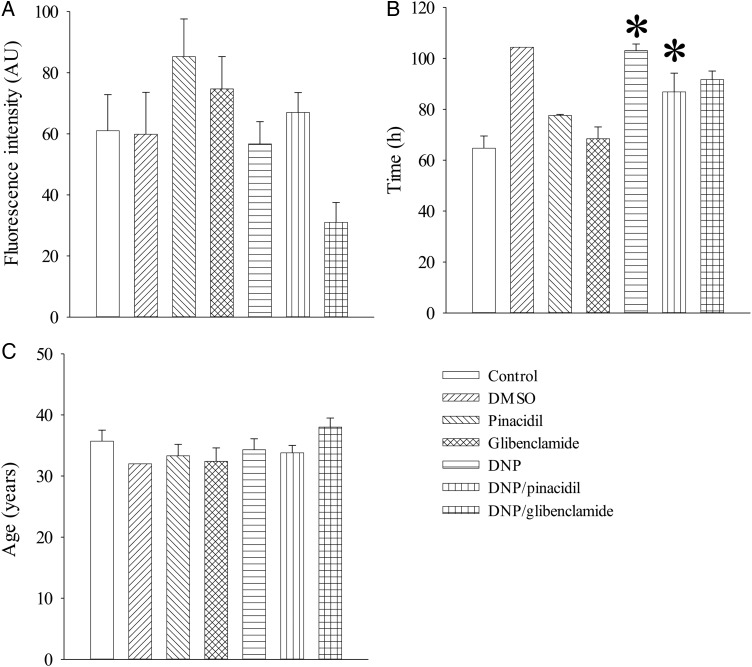
Age of donors, time between oocyte collection and experiments and basal level of Ca^2+^. Basal levels of intracellular Ca^2+^ in human oocytes and age of donors of oocytes was not different between control and experimental groups while the time between oocyte collection and experiments was longer only in the DNP and DNP/pinacidil experimental groups when compared with the control. Average fluorescence at time point 0 (**A**), time passed between oocytes collection and experiment (**B**) and age of donors of oocytes (**C**) for control and experimental groups. Bars show the mean ± SEM (*n* = 1–7 for age and 5–23 for basal fluorescence and time passed between oocytes collection and experiment). *P < 0.05, ANOVA. AU, arbitrary units.

All experiments were performed on supernumerary human mature (MII) oocytes. Oocytes were collected from 41 healthy women (average age 34.1. ± 0.6 years) undergoing assisted reproduction treatment at Ninewells Assisted Conception Unit, Dundee, UK [HFEA centre # 0004]. The main reasons for infertility in donors were unexplained (38%) and male factor (29%), while only 3% had endometriosis, a condition that could potentially affect Ca^2+^ homeostasis in oocytes (Andrade *et al.*, 2010; Carvalho *et al.*, 2012; Singh *et al.*, 2013). Ethical approval was provided by East of Scotland Research Ethics Service [number 08/S1402/23] and written informed consent was obtained from all women. MII oocytes used for this research had been inseminated (IVF) or injected (ICSI) for clinical treatment, but had failed to fertilize (no pronuclei and no cleavage). Oocytes were stripped for ICSI using ICSI Cumulase (Origio, Malov, Denmark), while oocytes for IVF were not stripped but at fertilization check remaining cells were mechanically removed. From the point of assessment of fertilization to being donated for research, all oocytes were maintained in 20 µl drops of cleavage medium under oil (SAGE; Cooper Surgical, Trimbull, CT, USA) at 37°C in 5% O_2_, 6%CO_2_ balanced with nitrogen. Oocytes were arbitrarily divided between experimental groups that were studied independently from each other in order to assess Ca^2+^ levels: (i) Control (in the absence of any compound expected to influence intracellular Ca^2+^), (ii) Dimethyl sulfoxide (DMSO; at 0.1% DMSO, as used to dissolve pinacidil, glibenclamide and 2,4-dinitrophenol (DNP)), (iii) Pinacidil (100 µM pinacidil, a K_ATP_ channel opener), (iv) Glibenclamide (100 µM glibenclamide, a K_ATP_ channel blocker), (v) DNP (100 mM DNP, an inhibitor of oxidative phosphorylation and inducer of chemical hypoxia), (vi) Pinacidil and DNP (both at 100 µM) and (vii) Glibenclamide and DNP (both at 100 µMs). Unless indicated differently, cells in each experimental group were monitored continuously for 2 h. In a separate series of experiments, oocytes were loaded with Fluo-3 at 37°C. These oocytes were also used to make comparison between those exposed to IVF or ICSI. Pinacidil, glibenclamide, DNP and DMSO were purchased from Sigma-Aldrich, Gillingham, UK. There was no statistically significant difference in basal levels of intracellular Ca^2+^ between experimental groups (Fig. 1). Time from oocyte retrieval to start of Ca^2+^ monitoring was not significantly different for the control group compared with pinacidil and glibenclamide treatment groups (Fig. 1). Female age of oocyte donors was also not significantly different between experimental groups (Fig. 1).

### Laser confocal microscopy and experimental protocol

MII oocytes for all experimental groups, apart from controls loaded at 37°C, were loaded (for 60 min) at room temperature with the esterified form of the Ca^2+^-sensitive fluorescent probe, Fluo-3AM (5 μM dissolved in 0.1% DMSO plus pluronic acid; Molecular Probes, Eugene, OR, USA). Afterwards, cells were superfused with collection solution (Sigma-Aldrich, M0393) with and without compounds targeting K_ATP_ channels (described in previous section) and imaged using laser confocal microscopy coupled to an inverted microscope (Leica TCS SP5 II, Milton Keynes, UK) with a ×10 (numerical aperture 1·3) oil-immersion objective lens at 37°C. The intensity of fluorescence of whole oocytes on the equatorial plane was measured. Microscope was calibrated by green calibration slide before each experiment. Intensity of fluorescence was described in arbitrary units (AU) covering a range from 0 to 60 000 AU. Ca^2+^ levels and cell morphology were imaged every 10 min for 2 h using an Argon/UV laser (excitation 488 nm/emission 520 nm). Images were analysed using Leica Application Suite AF Lite software (Leica). The parameters of image acquisition were similar for all examined cells. All compounds were purchased from Sigma-Aldrich. All obtained results were normalized in respect to the intensity of fluorescence at time point 0, that was considered to be 100%.

### Statistical analysis

Data are presented as mean ± SEM. Mean values were compared using analysis of variance (ANOVA), ANOVA on ranks and the paired or unpaired *t*-test, where appropriate. *P* < 0.05 was considered statistically significant. Statistical analysis was performed using SigmaPlot 12 (Systat Software, Hounslow, UK).

## Results

### Increase of Ca^2+^ *in vitro* over time in human oocytes

**Figure 2:**
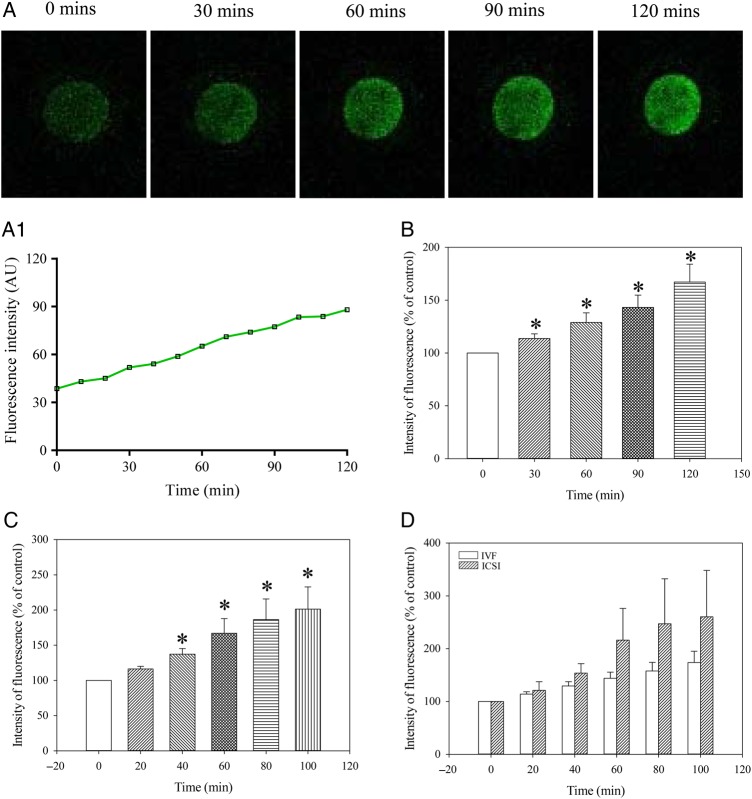
Ca^2+^ increases over time in MII human oocytes. (**A**) Laser confocal images from an untreated oocyte (magnification ×10) at depicted time points. (**A1**) Changes in intensity of Fluo-3 fluorescence plotted as a function of time from oocytes in Fig. 2A. (**B**) Normalized intensity of Fluo-3 fluorescence in condition/time points corresponding to (A). Bars are mean ± SEM (*n* = 19 oocytes). *P < 0.05. Normalized intensity of Fluo-3 fluorescence in oocytes loaded with Fluo-3 at 37C (**C**) and oocytes that were used for IVF or ICSI (**D**). Bars are mean ± SEM (*n* = 7–23 oocytes). *P < 0.05 (ANOVA and *t*-test).

**Figure 3:**
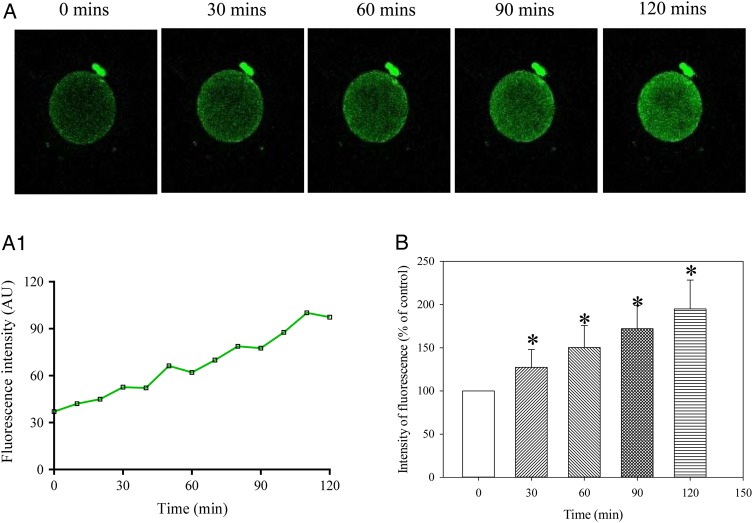
DMSO does not modify Ca^2+^ increases over time in MII human oocytes. (**A**) Laser confocal images from an oocyte (imaged at cation ×10) at depicted time points in the presence of DMSO (vehicle used to dissolve all compounds) (0.1%). (**A1**) Changes in intensity of Fluo-3 fluorescence plotted as a function of time from oocytes in Fig. 3A. (**B**) Normalized intensity of Fluo-3 fluorescence in condition/time points corresponding to (A). Bars are mean ± SEM (*n* = 8 oocytes). *P < 0.05 (ANOVA).

Ca^2+^ levels were captured every 10 min for 2 h. The increase in intensity of fluorescence reached statistical significance after 30 min (it increased by 13.7 ± 4.5% from the basal level), *n* = 19, *P* = 0.007; Fig. 2. After 120 min, intensity of fluorescence was increased by 67.2 ± 16.9% from the basal level (*n* = 19; *P* < 0.001 compared with time 0; Fig. 2). Similar results were obtained when cells were loaded at 37°C (Fig. 2C). We have also examined whether there was a difference in fluorescence intensity among oocytes obtained from IVF versus ICSI patients. The mean change in fluorescence observed between the two sets of data was not statistically significant (Fig. 2D). DMSO (0.1%) did not have any effect on dynamics of intracellular Ca^2+^ under *in vitro* conditions (Fig. 3).

### Pinacidil inhibits the increase in Ca^2+^ over time *in vitro* in human oocytes

**Figure 4:**
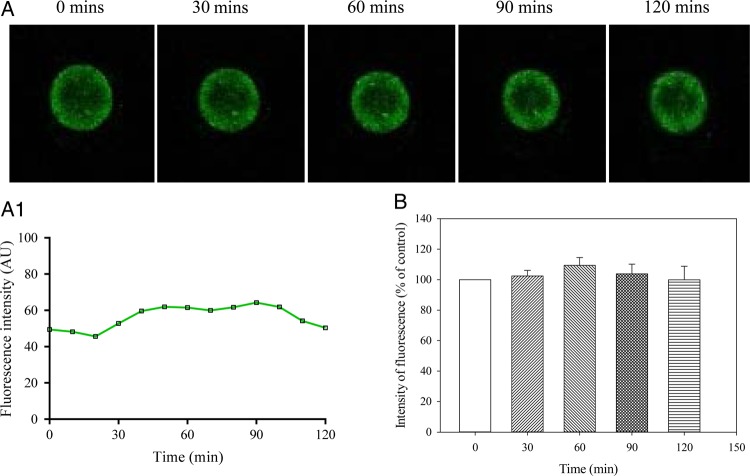
Ca^2+^ does not increase over time in MII human oocytes in the presence of pinacidil. (**A**) Laser confocal images from an oocyte (magnification ×10) in the presence of pinacidil (100 µM) at depicted time points. (**A1**) Changes in intensity of Fluo-3 fluorescence plotted as a function of time from oocytes in Fig. 4A. (**B**) Normalized intensity of Fluo-3 fluorescence in condition/time points corresponding to (A). Bars are mean ± SEM (*n* = 12 oocytes).

In the presence of 100 µM pinacidil, intracellular Ca^2+^ levels remain unchanged over time (*P* = 0.536; *n* = 12–19; Fig. 4). After 120 min, the intensity of fluorescence was 99.9 ± 8.9% of that at time point 0 (*P* = 0.993; *n* = 12–19; Fig. 4).

### Glibenclamide inhibits the increase in Ca^2+^ over time *in vitro* in human oocytes

**Figure 5:**
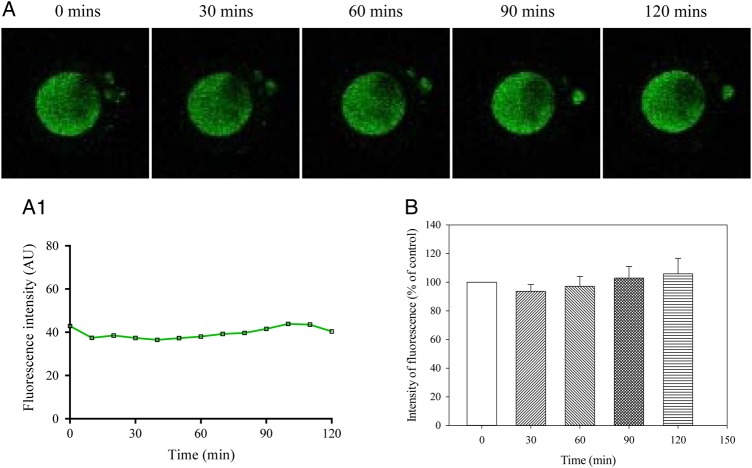
Ca^2+^ does not increase over time in MII human oocytes in the presence of glibenclamide. (**A**) Laser confocal images from an oocyte (magnification ×10) in the presence of glibenclamide (100 µM) at depicted time points. (**A1**) Changes in intensity of Fluo-3 fluorescence plotted as a function of time from oocytes in Fig. 5A. (**B**) Normalized intensity of Fluo-3 fluorescence in condition/time points corresponding to (A). Bars are mean ± SEM (*n* = 13 oocytes).

In the experimental group treated with glibenclamide (100 µM), no statistically significant changes in intracellular Ca^2+^ were observed (*P* = 0.762; *n* = 13; Fig. 5). After 120 min, the intensity of fluorescence was increased by only 5.9 ± 10.9% (*n* = 13; *P* = 0.600 when compared with fluorescence at the time point 0; Fig. 5).

### Ca^2+^ increases in human oocytes exposed to DNP

**Figure 6:**
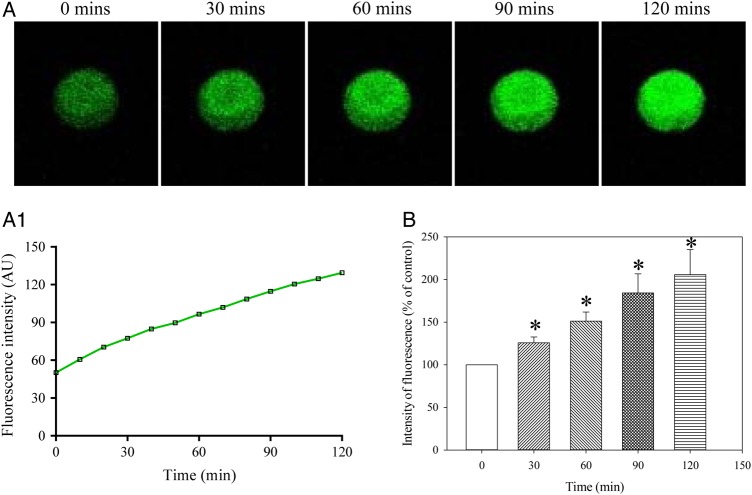
Ca^2+^ increases in MII human oocytes exposed to DNP. (**A**) Laser confocal images from an oocyte (magnification ×10) exposed to DNP (100 µM) at depicted time points. (**A1**) Changes in intensity of Fluo-3 fluorescence plotted as a function of time from oocytes in Fig. 6A. (**B**) Average intensity of Fluo-3 fluorescence in condition/time points corresponding to (A). Bars are mean ± SEM (*n* = 10 oocytes). *P < 0.05 (ANOVA).

DNP, an inhibitor of oxidative phosphorylation (Brady *et al.*, 1996), was used to assess intracellular Ca^2+^ dynamics in human oocytes when exposed to severe metabolic stress. Intracellular Ca^2+^ was significantly increased in the presence of 100 mM DNP (Fig. 6). Intensity of fluorescence increased by 25.9 ± 6.7 and 105.7 ± 29.5% after 30 and 120 min, respectively (*n* = 10; *P* = 0.004 for both time points when compared with fluorescence at time point 0; Fig. 6).

### DNP induces increase in Ca^2+^ in human oocytes in the presence of pinacidil

**Figure 7:**
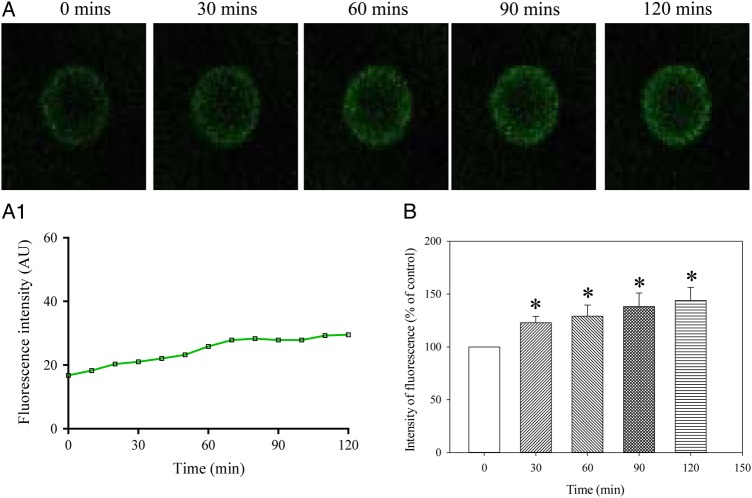
DNP induces an increase in Ca^2+^ in MII human oocytes despite presence of pinacidil. (**A**) Laser confocal images from an oocyte (magnification ×10) exposed to DNP (100 µM) plus pinacidil (100 µM) at depicted time points. (**A1**) Changes in intensity of Fluo-3 fluorescence plotted as a function of time from oocytes in Fig. 7A. (**B**) Normalized intensity of Fluo-3 fluorescence in condition/time points corresponding to (A). Bars are mean ± SEM (*n* = 5 oocytes). *P < 0.05.

DNP increased intracellular levels of Ca^2+^ despite the presence of 100 µM pinacidil (*n* = 5; *P* = 0.012; Fig. 7).

### Glibenclamide abolishes the increase in Ca^2+^ in human oocytes exposed to DNP

**Figure 8:**
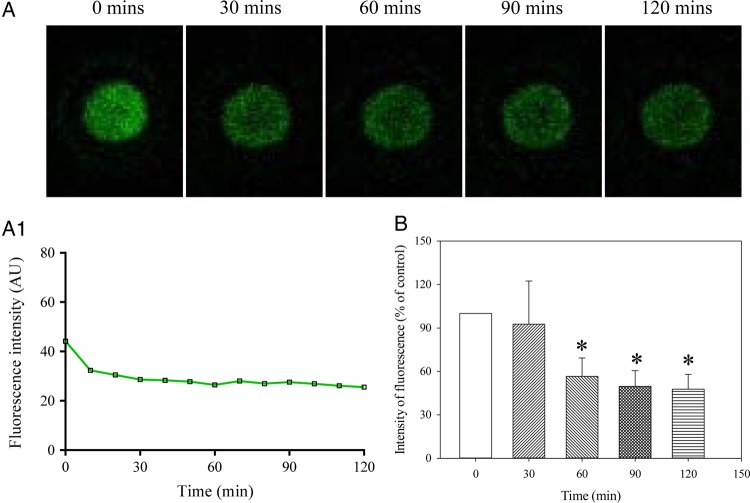
Glibenclamide abolishes the DNP-induced increase in Ca^2+^ in MII human oocytes. (**A**) Laser confocal images from an oocyte (magnification ×10) exposed to DNP (100 µM) plus glibenclamide (100 µM) at depicted time points. (**A1**) Changes in intensity of Fluo-3 fluorescence plotted as a function of time from oocytes in Fig. 8A. (**B**) Normalized intensity of Fluo-3 fluorescence in condition/time points corresponding to (A). Bars are mean ± SEM (*n* = 9 oocytes). *P < 0.05 (ANOVA).

In the presence of glibenclamide (10 µM), DNP (100 mM) did not increase the intracellular level of Ca^2+^ (Fig. 8). On the contrary, the intensity levels of fluorescence were significantly lower over the time course of 2 h, as it decreased by 52.3 ± 10.3% (*P* < 0.001; *n* = 9 for each; Fig. 8).

## Discussion

This study demonstrates that an increase in intracellular Ca^2+^ does occur in human oocytes when exposed to *in vitro* conditions and that this can be reduced and/or prevented by drugs targeting K_ATP_ channels.

IVF and ICSI are standard interventions aimed to treat infertility. In order to carry out these techniques, it is necessary to remove an oocyte from its natural environment (the follicle) and prepare it for further procedures *in vitro.* Although in handling and manipulation of the oocytes in the laboratory, we aim to minimize external stresses, data from work on oocytes of experimental animals and some other cell types indicate that cellular stress could occur (Martín-Romero *et al.*, 2008; Lord and Aitken, 2013). Due to clinical and ethical considerations, we were not able to monitor intracellular Ca^2+^ immediately after MII oocytes were collected as they were intended for therapeutic use and we obtained them only after IVF or ICSI were deemed unsuccessful. On average, cells were obtained for experimentation 80.4 ± 2.1 h (*n* = 102 oocytes) after collection, so we do not have information about Ca^2+^ dynamics prior to this period.

In this study, we have demonstrated a time-dependent increase in intracellular Ca^2+^ during monitoring of MII human oocytes for 2 h in *in vitro* conditions. This is the first report to suggest that Ca^2+^ homeostasis is challenged when oocytes are incubated under routine *in vitro* conditions, although this might be associated solely with protracted incubation. During incubation of oocytes, we have maintained conditions similar to physiological ones. The only exception was loading of cells with Ca^2+^-sensitive dye at room temperature. It has been previously reported that drops in temperature cause depolymerization of microtubules with increased risk of segregation errors and aneuploidy. Depolymerization of microtubules is influenced by Ca^2+^ (Aman and Parks, 1994). However, loading of cells with Ca^2+^ at 37°C did not increase the intracellular Ca^2+^ under *in vitro* conditions, suggesting that temperature of the Ca^2+^-sensitive dye did not affect Ca^2+^ dynamics. Subfertile women with endometriosis have elevated levels of systemic and intrafollicular oxidative stress markers (Andrade *et al.*, 2010; Carvalho *et al.*, 2012; Singh *et al.*, 2013), thus oocytes arising from such patients could have potentially elevated intracellular Ca^2+^. However, in the present study the overwhelming majority of donors were healthy, excluding the possibility that the oocytes we studied had altered Ca^2+^ homeostasis due to pathological process. It has been reported that ICSI is less successful in bovine and equine species due to altered Ca^2+^ dynamics in response to injection (Bedford *et al.*, 2003; Malcuit *et al.*, 2006). In addition to that, a difference in intracellular Ca^2+^ response was observed (Markoulaki *et al.*, 2007) between oocytes subjected to ICSI or IVF procedures. Therefore, it was possible that oocytes subjected to ICSI or IVF respond differently to *in vitro* conditions. However, that was not the case as we did not find a statistically significant difference in Ca^2+^ dynamics between the two groups. Taken all together, our findings suggest that an increase in Ca^2+^ in oocytes persistently occurs under *in vitro* conditions.

Ca^2+^ is a crucial signal for oocyte fertilization, as well as for early embryo development, and any adverse changes in Ca^2+^ homeostasis could feasibly decrease the probability of successful conception by a negative impact on oocyte quality (reviewed by Whitaker, 2006). This could represent an underlying reason for failure of IVF or ICSI. It seems that the duration of time that oocytes spending under *in vitro* conditions is an important factor determining success fertilization. Also, it has been reported that mice generated by IVF suffer from vascular dysfunction later in life and have a shortened lifespan (Rexhaj *et al.*, 2013). As Ca^2+^ is a signalling molecule regulating a range of cellular functions (Clapham, 2007), it is possible that even when conception occurs it may have consequences for later life. There is no visible manifestation of increased Ca^2+^ in oocytes and the issue is that a Ca^2+^-overloaded oocyte may be selected/used for IVF/ICSI resulting either in failure of fertilization or successful fertilization associated with health issues occurring later.

**Figure 9:**
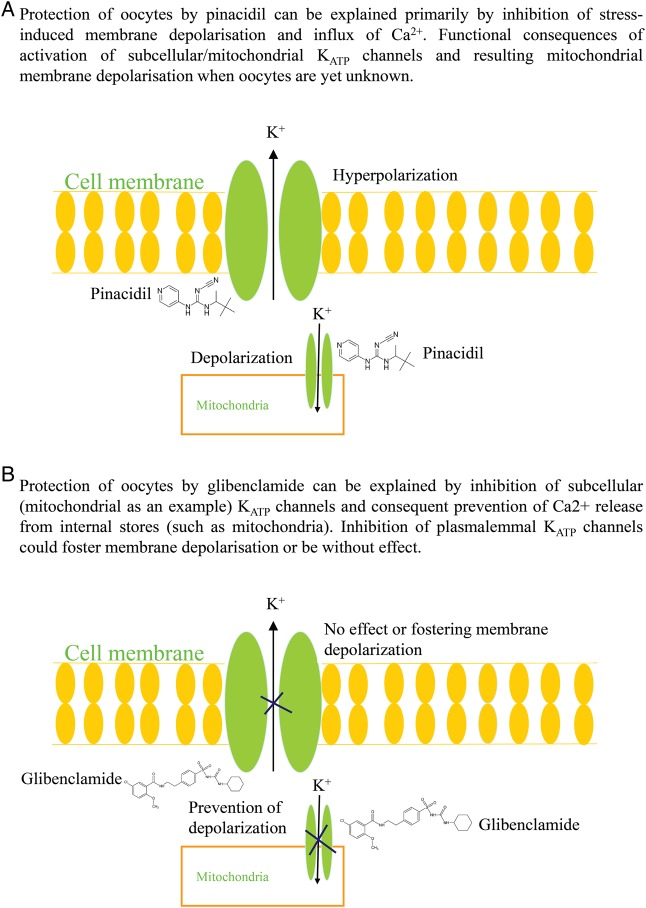
A possible mechanism underlying cytoprotective action of pinacidil and glibenclamide in human oocytes. Cartoon summarizing possible underlying mechanisms mediating pinacidil- (**A**) and glibenclamide-induced (**B**) cytoprotection in MII human oocytes based on the findings from the present study as well as findings from previous studies that have investigated K_ATP_ channels in oocytes and other cell types (Brady *et al.*, 1996; Holmuhamedov *et al.*, 1998; Jovanović *et al.*, 1999; Jovanović and Jovanović, 2001; Du *et al.*, 2010).

It has been recently shown that oocytes express K_ATP_ channels (Du *et al.*, 2010). K_ATP_ channels link intracellular metabolic conditions with the membrane excitability (Nichols, 2006). We have hypothesized that activation of K_ATP_ channels could prevent Ca^2+^ loading in oocytes occurring *in vitro*. Therefore, we have tested the effect of pinacidil, a well-established K_ATP_ channel-opening drug (Nichols, 2006). In the presence of pinacidil, Ca^2+^ levels remained steady suggesting that the activation of K_ATP_ channels prevents Ca^2+^ loading induced by *in vitro* conditions. It has been shown that the opening of K_ATP_ channels clamps the membrane potential at a more negative value, keeping voltage-dependent Ca^2+^ entry pathways closed and preventing influx of Ca^2+^ (Jovanović and Jovanović, 2001). Taking into consideration that influx of Ca^2+^ is a crucial part of Ca^2+^ signalling in mammalian oocytes (reviewed by Gómez-Fernández *et al.*, 2012), the observed inhibition of Ca^2+^ loading in human oocytes is in agreement with such an effect of K_ATP_ channel opening. On the other hand, glibenclamide is an oral antidiabetic known to inhibit K_ATP_ channels opening (reviewed by Abdelmoneim *et al.*, 2012). In cells where K_ATP_ channels are physiologically closed application of this drug usually has no significant effects (Brady *et al.*, 1996; Budas *et al.*, 2004), while in cells where K_ATP_ channels are open, glibenclamide often induces membrane depolarization and Ca^2+^ loading, even leading to cell death (Brady *et al.*, 1996; Budas *et al.*, 2004; Abdelmoneim *et al.*, 2012). Surprisingly, we have found that glibenclamide actually prevents Ca^2+^ loading induced by *in vitro* conditions in human MII oocytes. Structurally, K_ATP_ channels are composed of a pore-forming inward rectifier, Kir6.1 or Kir6.2, and a regulatory, ATP-binding subunit, SUR1, SUR2A or SUR2B. It is generally accepted that four Kir6.x and four SURx are physically associated with each other to form functional K_ATP_ channels (reviewed by Nichols, 2006). In our previous study, we have suggested that the K_ATP_ channel composition in human MII oocytes is the SUR2A/Kir6.1 combination (Du *et al.*, 2010). Kir6.1 is a pore-forming channel subunit in the vascular form of K_ATP_ channels (Nichols, 2006). Glibenclamide normally increases intracellular Ca^2+^ (Brady *et al.*, 1996; Budas *et al.*, 2004; Abdelmoneim *et al.*, 2012). The only finding similar to ours was reported on vascular smooth muscle cells where glibenclamide inhibited the increase in Ca^2+^ evoked by high extracellular K^+^ and noradrenaline (Yoshitake *et al.*, 1991) despite inducing membrane depolarization (Wilson *et al.*, 2000). It is certainly intriguing that compounds inhibiting and activating K_ATP_ channels can act in the same manner to prevent Ca^2+^ loading induced by *in vitro* conditions. One possible explanation is that prevention of the Ca^2+^ increase is due to the differential effects of pinacidil and glibenclamide on different populations of K_ATP_ channels in oocytes. As an example, the activation of plasmalemmal K_ATP_ channels prevents Ca^2+^ influx by clamping the membrane potential (Jovanović and Jovanović, 2001), but the inhibition of K_ATP_ channels localized in mitochondria inhibits release of their Ca^2+^ (Holmuhamedov *et al.*, 1998). Thus, it is quite plausible that a decrease in Ca^2+^ induced by pinacidil is due to inhibition of membrane depolarization, while a decrease in Ca^2+^ induced by glibenclamide could be due to inhibition of mitochondrial release of Ca^2+^ (Fig. 9).

DNP is an inhibitor of oxidative phosphorylation. It induces severe metabolic stress (Brady *et al.*, 1996; Du *et al.*, 2010), but also activates K_ATP_ channels in human oocytes (Du *et al.*, 2010). When exposed to DNP, intracellular levels of Ca^2+^ increased in human oocytes, but this increase was not dramatically different to those observed when oocytes were left under *in vitro* conditions alone. It is possible that either (i) DNP has no a major effect on human oocytes or (ii) that human oocytes had passed a threshold of *in vitro* stress. In pig oocytes, exposure to *in vitro* conditions alone does not affect intracellular levels of Ca^2+^, while exposure to DNP significantly increases intracellular Ca^2+^ (unpublished data) suggesting that DNP induces significant stress in mammalian oocytes (Grundlingh *et al.*, 2011). We conclude that the inability of DNP to further increase Ca^2+^ in human oocytes is probably due to the already high sensitivity and stress of these cells following extended *in vitro* conditions. Pinacidil inhibited Ca^2+^ loading induced solely by *in vitro* conditions, but was not as efficient on DNP-induced Ca^2+^ loading. In contrast, glibenclamide maintained Ca^2+^ homeostasis in oocytes despite cells being challenged by DNP. It seems that glibenclamide, an antagonist of K_ATP_ channels, is more efficient as cytoprotective agent in human oocytes than pinacidil, a K_ATP_ channel opener.

In principle, compounds that protect oocytes against stress occurring in *in vitro* conditions have a potential to improve the rate of fertilization *in vitro* and embryo quality. This study suggests that compounds targeting K_ATP_ channels should be tested as a means for improving the outcome of ART.

## Conclusion

In conclusion, this study has demonstrated that human oocytes maintain Ca^2+^ homeostasis with difficulty when exposed to routine *in vitro* conditions, which could interfere with fertilization. Both inhibition and activation of K_ATP_ channels is useful for maintaining Ca^2+^ homeostasis in oocytes under *in vitro* conditions. It seems that inhibition of K_ATP_ channels is a particularly efficient strategy in protecting human oocytes against stress. Such a strategy should be tested in an ART setting to examine if it leads to an improve rate of success.

## Authors' roles

All authors have contributed to the study design, revised and approved the manuscript. In addition to that, G.F., N.D., N.K., A.M., M.G., E.D., E.B., O.A.M., S.G.B., A.M.M., S.J.M.S. and K.M.W. were involved in oocyte and patients data collection, whilst G.F., N.D., N.K., A.M. and A.M.M. have performed experiments using these oocytes. A.J. has designed and supervised the study, drafted and approved the manuscript.

## Funding

University of Dundee, MRC (MR/K013343/1, MR/012492/1), NHS Tayside, Funding NHS fellowship (Dr Sarah Martins da Silva), NHS Scotland. Funding to pay the Open Access publication charges for this article was provided by MRC.

## Conflict of interest

None declared.
